# What are the attitudes of health professionals regarding patient reported outcome measures (PROMs) in oncology practice? A mixed-method synthesis of the qualitative evidence

**DOI:** 10.1186/s12913-020-4939-7

**Published:** 2020-02-10

**Authors:** Bróna Nic Giolla Easpaig, Yvonne Tran, Mia Bierbaum, Gaston Arnolda, Geoff P. Delaney, Winston Liauw, Robyn L. Ward, Ian Olver, David Currow, Afaf Girgis, Ivana Durcinoska, Jeffrey Braithwaite

**Affiliations:** 1 Australian Institute of Health Innovation, Macquarie University, North Ryde, NSW 2109 Australia; 2 Liverpool Cancer Therapy Centre, Liverpool, NSW 2170 Australia; 3 South Western Sydney Clinical School, University of New South Wales, Liverpool, NSW 2170 Australia; 4 Centre for Oncology Education and Research Translation (CONCERT), Ingham Institute for Applied Medical Research, Liverpool, NSW 2170 Australia; 5 St. George Cancer Care Centre, St. George Hospital, Kogarah, NSW 2217 Australia; 6 St. George Hospital Clinical School, University of New South Wales, Sydney, NSW 2217 Australia; 7 Faculty of Medicine and Health, The University of Sydney, Sydney, NSW 2006 Australia; 8 Prince of Wales Clinical School, University of New South Wales, Sydney, NSW 2052 Australia; 9 Faculty of Health and Medical Sciences, University of Adelaide, Adelaide, SA 5000 Australia; 10 College of Medicine and Public Health, Flinders University, Adelaide, SA 5042 Australia; 11 Faculty of Health, University of Technology Sydney, Sydney, NSW 2007 Australia

**Keywords:** Oncology, Patient reported outcome measures, PROMs, Health professional attitudes, Qualitative synthesis, Implementing practice change

## Abstract

**Background:**

The adoption of Patient Reported Outcome Measures (PROMs) in cancer care has been widely advocated, but little is known about the evidence for the implementation of PROMs in practice. Qualitative research captures the perspectives of health professionals as end-users of PROMs and can be used to inform adoption efforts. This paper presents a systematic review and synthesis of qualitative research conducted to address the question: What are the attitudes of health professionals towards PROMs in oncology, including any barriers and facilitators to the adoption of PROMS, reported in qualitative evidence?

**Methods:**

Systematic searches of qualitative evidence were undertaken in four databases and reviewed using the Preferred Reporting Items for Systematic Reviews and Meta-Analyses guidelines. Studies published in English between 1998 and 2018, which reported qualitative findings about the attitudes of health professionals working in oncology towards PROMs were eligible. Studies were assessed using the Critical Appraisal Skills Programme’s Qualitative Research Checklist. A sentiment analysis was conducted on primary text to examine the polarity (neutral, positive or negative) of health professionals’ views of PROMs. Qualitative meta-synthesis was conducted using a constant comparative analysis.

**Results:**

From 1227 articles after duplicates were removed, with 1014 excluded against the screening criteria, 213 full text articles remained and were assessed; 34 studies met the inclusion criteria and were included. The majority of studies were of good quality. Sentiment analysis on primary text demonstrated an overall positive polarity from the expressed opinions of health professionals. The meta-synthesis showed health professionals’ attitudes in four domains: identifying patient issues and needs using PROMs; managing and addressing patient issues; the care experience; and the integration of PROMs into clinical practice.

**Conclusions:**

From the accounts of health professionals, the fit of PROMs with existing practice, how PROMs are valued, capacity to respond to PROMs and the supports in place, formed the key factors which may impede or promote adoption of PROMs in routine practice. To assist policy-makers and services involved in implementing these initiatives, further evidence is required about the relationship between PROMs data collection and corresponding clinical actions.

**Trial registration:**

International Prospective Register of Systematic Reviews (PROSPERO) CRD42019119447, 6th March, 2019.

## Background

Promoting patients’ engagement with their health care has been viewed as a means of improving the identification of patient needs and priorities and creating opportunities to address those needs during the cancer journey [1, 2]. Patient-reported outcome measures (PROMs) are derived from patient self-assessment of a variety of health and wellbeing indices, and provide information to health professionals (HPs) about the patient’s health status [3, 4]. PROMs data may relate to one or multiple health-relevant domains including psychological and physical wellbeing, and be collected using a range of electronic and/or paper-based mediums [5]. Studies have identified differences between patient and clinician assessments of outcomes in oncology with regard to treatment side effects, numerous physical symptoms, as well as psychological issues, whereby oncologists only identified a small proportion of the total patients who were experiencing clinical anxiety and depression [6, 7]. This discordance has not improved over the past two decades [6, 7], supporting the need for patients’ direct reports. A systematic review found that PROMs may be useful in cancer care, to longitudinally monitor and respond to the impacts of treatments or symptoms on patients’ lives [8]. For example, some PROMs are designed to automatically trigger the provision of tailored information to patients to help them address their symptoms and side-effects [4, 9, 10].

Systematic collection and feedback of PROMs results to the care team is reported to improve processes and outcomes of care [1, 4]. In a randomised controlled trial, 766 patients receiving outpatient chemotherapy were randomly assigned to an intervention or control group. The control group received standard care with symptoms monitored by the treating clinician while the intervention group, in addition, electronically reported on 12 common symptoms at set times [1]. Patients in the intervention group scored higher on the health-related quality of life measure, showed greater treatment adherence, had fewer hospital admissions and had a higher survival rate at 1 year. Ahmed and colleagues [11] propose that PROMs are useful for comparing treatments and can also be used to evaluate quality improvement activities. Additionally, a systematic review of the effects of PROMs on clinical practice identifies potential benefits in micro-level patient-clinician interactions, predominantly by enhancing communication and revealing psychological and physical issues [5, 12]. However, evidence for the impact of PROMs on clinical practices, such as prompting appropriate referrals, is reported to be weak [5, 13] or ambiguous [8], and a greater understanding of how PROMs may be integrated and used in clinical care has been sought [3, 5].

Howell et al. [3] observed that little is known about the evidence concerning the introduction of PROMs into routine practice. A strength of qualitative research is that it captures the perspectives of those involved in interventions or programs, such as during the introduction of PROMs. This information may help to guide the future implementation [14]. Boyce and colleagues [15] undertook a systematic review of qualitative research that examined HPs’ views and experiences with PROMs, through which they identified a set of concerns. HPs raised practical concerns about possible increases in workload, especially where PROMs were not fully integrated into existing patient management systems, and highlighted the importance of training. Some HPs were not open to changing their practices and harboured negative attitudes towards PROMs, potentially hindering adoption. In some studies, clinicians suggested that the relative clinical importance of different PROMs needed clarification, and PROMs data needed to be aggregated to contextualise and complement other clinical data. Mixed views were reported about the capacity of PROMs to improve patient care and some clinicians worried about negative impacts on the patient-clinician relationship. On the positive side, the Boyce review [15] reported that professionals believe PROMs increased patient education, stimulated better care planning and built confidence in the competence of the professional.

The Boyce et al. [15] review raised a number of important issues, but excluded qualitative research from mixed-methods studies. Such studies are often used in program development and studies of acceptability and feasibility. While some of the studies examined were in oncology (*n* = 1) and palliative care (*n* = 5), the focus of that review was not disease specific. Extending previous work, the present systematic review and synthesis of qualitative research concerning the attitudes of HPs to PROMs, specific to cancer care, will provide insights to guide implementation efforts.

### Review question

What are the attitudes of HPs towards PROMs in oncology, including any barriers and facilitators to the adoption of PROMs, reported in qualitative evidence?

## Methods

### Search strategy

Search strategies were adopted from Boyce et al. [15], revised to reflect updated terminology and the oncology focus. The strategy contained five blocks of relevant terms and keywords for: 1) patient-reported outcomes, 2) qualitative research, 3) attitudes, 4) HPs, and 5) oncology. Medline, Cinahl, Embase and PsychInfo databases were searched in October 2018, and updated in April 2019 to capture literature published from January 1998 to December 2018. Results of the searches were imported into EndnoteX9 [16] and duplicates removed. Reference lists of included papers were screened. The protocol was registered with Prospero (no. CRD42019119447).

### Study inclusion criteria

**Fig. 1:**
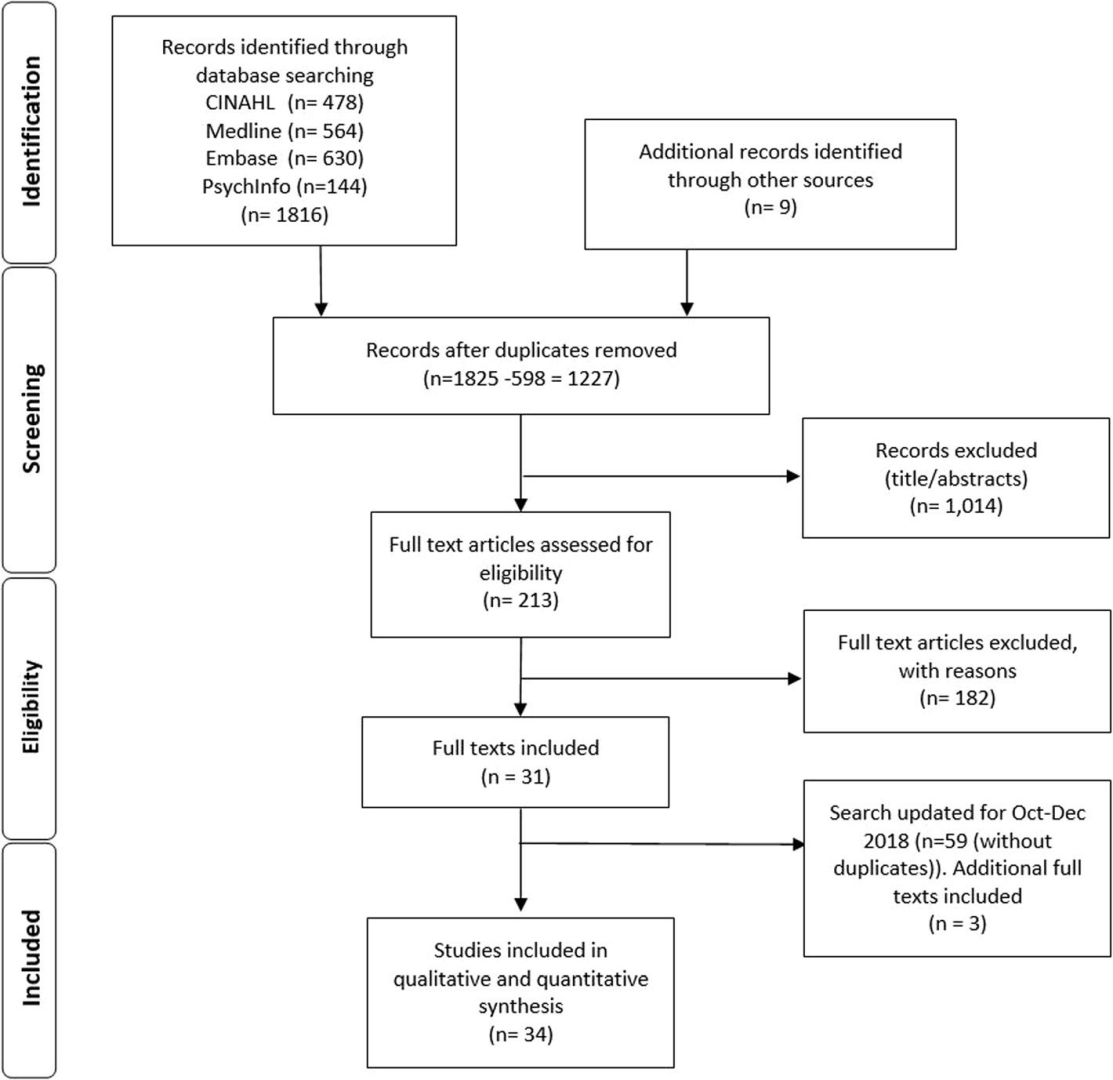
PRISMA [17] flowchart of search strategy

Studies were included if they were: 1) qualitative or mixed-method, where the qualitative data was analysed and reported separately; 2) published in English and reported primary findings; and 3) reported attitudes (broadly defined to include views, perceptions and perspectives) of HPs working in oncology towards PROMs. Study selection was documented and is summarised in a Preferred Reporting Items for Systematic Reviews and Meta-Analyses (PRISMA)-compliant flow chart [17](see Fig. 1; Additional file 1).

### Study selection

Four independent reviewers (BNGE, MB, YT, GA) each reviewed a quarter of the titles and abstracts. A random selection of 5% of title abstracts were jointly reviewed to determine inter-rater reliability. Full text was retrieved for title abstracts which appear to meet inclusion criteria, and the article assessed independently by at least two team members to determine eligibility. Disagreements were resolved by team consensus.

### Study quality assessment

The quality of studies was assessed using the Critical Appraisal Skills Programme, Qualitative Research Checklist [18]. This tool was specifically designed for the assessment of a range of dimensions of quality in qualitative research. This includes items to help assess the “Risk of bias in individual studies” [17] by examining the extent to which researchers considered their potential influence and bias (e.g. “Has the relationship between researcher and participants been adequately considered?”), as well as examining the appropriateness of the methodology and study design in this regard [18].

### Data extraction strategy

A purpose-designed template was used to guide data extraction, which included: citation, year, title, location of study, study aim/s, study setting, study design, data collection method, cancer stream, description of PROMs reported, HP role (where reported), key barriers to PROMs use, key facilitators to PROMs use, key attitudes towards PROMS, and other details noted as important in the study.

### Data synthesis and presentation

#### Sentiment analysis

To assess whether the reported opinions and attitudes of HPs to PROMs were neutral, positive or negative, primary data from results sections were quantitatively text-mined and a sentiment analysis conducted. Sentiment analyses use natural language processing to computationally examine the opinions, sentiment and subjectivity in text [19]. Our sentiment analysis was conducted using R versions 3.5.2 and RStudio (v1.1.442) [20, 21]. Sentiment scoring was performed using the Sentimentr package, examining polarity of text by applying existing sentiment dictionaries and taking into account valence shifters in text such as words that act as negators and amplifiers (e.g. I am not very happy) [22]. Words that attracted a sentiment score, such as “patient” or “kind”, but which were not used in the context of a positive sentiment were excluded in the sentiment scoring. Polarity scores were generated for each sentence, with 0 representing a neutral sentence, a negative score representing a negative sentiment and a positive score represented a positive sentiment.

#### Thematic meta-synthesis

The extracted data for each area of focus (attitudes, barriers and facilitators) were aggregated and thematic meta-synthesis was performed [23, 24]. NVivo 12 software was used to manage and assist analysis [25]. In Stage 1, two team members (BNGE, MB) independently undertook line by line coding of subsets of data comprised of study results sections (i.e. including the raw data) and discussion sections (including the authors’ interpretations). The review question was sufficiently broad in scope to accommodate an inductive free coding approach. Each member independently developed a codebook on a subset of articles and these were reconciled through discussion to develop a single coding framework. In Stage 2, the framework guided the coding of all data to group codes into descriptive themes. Constant comparative analysis enabled the translation of concepts between studies. The framework was refined as needed during this process and provided a foundation for the development of analytical themes. An iterative process of engaging with the descriptive themes, the review question and raw data was undertaken in Stage 3. This resulted in the development of analytical themes, which while grounded in the data, provide insights and understandings that move beyond the data. Discrepancies were resolved by team discussion.

## Results

### Characteristics of included studies

The search conducted for the period January 1998 to October 2018 returned 1218 unique records. The review of the reference lists of included papers identified 9 additional records. The full text of 213 papers was reviewed and 182 papers were excluded on the basis that: 1) qualitative and quantitative findings were not reported separately; 2) it was not possible to distinguish findings from HPs from other groups (e.g. patients); and/or 3) PROMs were not a primary focus of study (i.e., they were captured incidentally). As a result, 31 papers were included. The search conducted for the period October–December 2018 returned 59 unique records which were screened and 56 were excluded for the reasons previously described. This resulted in the inclusion of an additional 3 papers. In total 34 papers were included.

Studies were most commonly conducted in the UK (*n* = 14), the USA (*n* = 9), and Australia (*n* = 3) and included professionals working in a range of settings, typically within clinics or treatment centres (*n* = 13). Ten studies specified the research was undertaken in acute care, while some studies were undertaken in a mixture of settings (see Table 1).

**Table 1:** Characteristics of studies

Study	Year	Location of study	Setting	Study design	Data collection method	Cancer stream	Participants	PROM name	Aims (verbatim)	*Was there a clear statement of the aims of the research?*	*Is a qualitative methodology appropriate?*	*Was the research design appropriate to address the aims of the research?*	*Was the recruitment strategy appropriate to the aims of the research?*	*Was the data collected in a way that addressed the research issue?*	*Has the relationship between researcher and participants been adequately considered?*	*Have ethical issues been taken into consideration?*	*Was the data analysis sufficiently rigorous?*	*Is there a clear statement of findings?*	*How valuable is the research?*
Quality assessment																			
Absolom et al. [26]	2011	UK	Hospitals (*n* = 3)	Qual	Interviews	Multiple	Doctors (oncologists and surgeons) (*n* = 12) and nurses (*n* = 11)	Various	“This study explores the views of cancer professionals regarding their current roles and responsibilities in the detection and management of ED, use of screening tools and access to expert psychological support”. (p.601)	Y	Y	Y	Y	Y	N	Y	Y	Y	Y
Basch et al. [27]	2005	USA	Cancer Centre in hospital	Qual	Survey and team debriefing session	Gyne-cological	Doctors (oncologists) (*n* = 5) and nurses (n = 4)	CTCAE (selected items), ECOG, EuroQoL EQ-5D and free text space	“By measuring patient and staff use of this system, two distinct but interrelated issues may be addressed: the feasibility of patient symptom self-reporting, and the usefulness of the Internet as a medium for PRO collection”. (p.3553)	Y	Y	Y	Y	Y	N	Y	U	Y	Y
Biddle et al. [28]	2016	UK	Centres (*n* = 2)	MM	Interviews	Multiple	Radiographers (*n* = 5) and nurses (*n* = 2)	DT and Problem List	“This qualitative study aimed to understand how such tools are experienced by patients and clinicians in order to optimise use in the future” (p.59)	Y	Y	Y	Y	Y	N	Y	Y	Y	Y
Brundage et al. [29]	2019	Canada	Cancer Centres (*n* = 4)	MM	Interviews	Prostate	Doctors (urologists and oncologists), and nurses (*n* = 31)	EPIC-CP and ESAS	“The specific study aims were to (1) evaluate the acceptability and usability of EPIC-CP through a patient ‘exit’ survey and (2) explore the clinicians’ acceptability of the EPIC-CP PRO data and use in clinical practice”. (p.772)	Y	Y	Y	Y	Y	N	Y	Y	Y	Y
Carolan and Campbell [30]	2016	UK	Primary care	Qual (Phenomenological)	Interviews	Multiple	General practitioners (*n* = 7)	Various	“This study sought to explore GPs’ experiences of assessing psychological distress in cancer patients across the cancer trajectory, including their use of validated screening tools.” (p.392)	Y	Y	Y	Y	Y	U	Y	Y	Y	Y
Cox et al. [31]	2011	UK	Hospices (*n* = 3)	MM	Interviews	Lung	Health professionals (*n* = 13)	ESAS and EuroQoL EQ-5D	“This study had two aims: (1) to test and evaluate the support provided to patients by the computerized assessment tool (the HealthHUBTM); and (2) to determine the clinical acceptability of the technology in a palliative care setting. This paper will focus on the clinical acceptability of the tool and present the difficulties in evaluating the support the tool provided to patients”. (p.676)	y	Y	Y	Y	Y	N	Y	Y	Y	Y
DuBenske et al. [32]	2008	USA	Cancer Centres (*n* = 5)	MM	Interviews	Multiple	Doctors (*n* = 4) and nurses (*n* = 3)	ESAS, Karnofsky Performance Scale and additional items	“This study reports initial findings from implementation of the Clinician Report (CR)—a patient and caregiver status report tool accessible by the oncology clinic team”. (p.679)	Y	Y	Y	Y	Y	N	Y	Y	Y	Y
Gamlen and Arber [33]	2013	UK	Community- based	Qual (Ethnographic)	Interviews and observations	Multiple	Nurses (*n* = 6)	Symptoms and Concerns Checklist	“The aim of the study is to explore how specialist cancer nurses carry out first assessments of patients in the community, their use of the Symptoms and Concerns Checklist (SCC) and their views on first assessments”. (p.797)	Y	Y	Y	Y	Y	Y	Y	Y	Y	Y
Girgis et al. [10]	2017	Australia	Hospitals (*n* = 2)	MM	Interviews	Multiple	Doctors (oncologists) *n* = (3), nurse (*n* = 1), and health services manager (*n* = 1)	DT, ESAS, Problem Checklist and SCNS-ST9	“The aim of this study was to test the feasibility and acceptability of PROMPT-Care (Patient Reported Outcome Measures for Personalized Treatment and Care)”. (p.1)	Y	Y	Y	Y	Y	N	Y	Y	Y	Y
Groff et al. [34]	2018	Canada	Cancer Clinics (*n* = 2)	MM	Interviews	Multiple	Doctors (oncologists) (*n* = 6), nurses (*n* = 7), and administrators (*n* = 3)	Canadian Problem Checklist and ESAS	“The purpose of this study was to evaluate the sustainability of an SFD program implemented in 2 separate oncology clinics 6 months following the conclusion of the implementation. This study also sought to shed light on the barriers and facilitators to the sustainability of SFD”. (p.142)	Y	Y	Y	Y	Y	Y	Y	Y	Y	Y
Handberg et al. [35]	2018	Denmark	Haematological wards in hospitals (*n* = 2) and primary Care settings (*n* = 2)	Qual (Interpretive Description, Ethnographic)	Semi-structured focus group interviews, observations, informal conversations and fieldnotes	Multiple	Nurses and allied health (*n* = 41)	Various	“The purpose of this study was to analyze and describe health professionals’ attitudes and perspectives on the complexities of cancer survivorship and rehabilitation needs assessment in a shared cancer care context”. (p.71)	Y	Y	Y	Y	Y	Y	Y	Y	Y	Y
Hubbard et al. [36]	2014	USA	Centre	MM	Survey	Multiple	Health professionals (*n* = 13 estimated)	SAQ	“We set out to determine the impact of PRO assessment on routine clinical practice”. (p.248)	Y	Y	Y	Y	Y	N	Y	U	Y	Y
Jagsi et al. [37]	2013	USA	Sample from various from practice settings	Qual	Interviews	Multiple	Doctors (oncologists) (*n* = 17)	Various	“In this study, we conducted semi-structured interviews to investigate practicing oncologists’ perceptions of PROs, with a focus on identifying the critical features to ensure acceptance of the collection of PROs within the context of routine oncology clinical practice”. (p.290)	Y	Y	Y	Y	Y	Y	Y	Y	Y	Y
Javid et al. [38]	2017	USA	Sample from various from practice settings	Qual	Web conference discussion and stakeholder panel	Breast	Doctors (surgeons) (*n* = 12)	Breast-Q	“We conducted an exploratory qualitative study in order to better understand what HRQOL domains and processes of care define high quality surgical care for women undergoing mastectomy for breast cancer from both the patient and clinician perspective”. (p.127)	Y	Y	Y	Y	Y	N	Y	U	U	Y
Kallen, et al. [39]	2012	USA	Palliative Care Clinic	MM	Interviews	Multiple	Doctors (*n* = 4) and nurses (*n* = 5)	Various	“Our project’s specific aims were to: (1) define a PRO-based palliative/hospice care model for use in developing the prototype software and (2) evaluate the prototype software in terms of the system’s usability and usefulness”. (p.168)	Y	Y	Y	U	Y	N	U	U	Y	Y
Kendall et al. [40]	2013	UK	Primary care (*n* = 13)	MM	Interviews	Multiple	Health Professionals (*n* = 29)	Cancer Ongoing Review Document	“This project aimed to assess the feasibility of early proactive follow-up in primary care using a structured template, from the perspective of patients with a new diagnosis of any cancer, their relatives and their primary care teams”. (p.303)	Y	Y	Y	Y	Y	Y	Y	Y	Y	Y
Kettis-Lindblad et al. [41]	2007	Sweden	Hospitals (*n* = 2)	Qual (Interpretivist)	Interviews	Multiple	Doctors (*n* = 6)	SEIQoL–DW and Disease-related SEIQoL-DW	“Overall, this study explored patients’ and oncologists’ perceptions of individualized QOL assessments—the SEIQoL Direct Weight (SEIQoL–DW) and the Disease-Related (DR) SEIQoL-DW—to support the consultation” (p.282)	Y	Y	Y	Y	Y	N	Y	Y	Y	Y
Korzeniowski et al. [42]	2016	Canada	Cancer Centre	Qual	Interviews	Prostate	Doctors (oncologists and a resident) (*n* = 6) and nurses (*n* = 4)	EPIC-26	“The purpose of this study was to pilot-test the use of EPIC-26 in a clinical context in order to assess the acceptability and added value of measuring EPIC-26 scores in practice from clinician and patient perspectives”. (p.1983)	Y	Y	Y	Y	Y	U	Y	Y	Y	Y
Kotronoulas et al. [43]	2017	UK	Hospitals (*n* = 3)	MM	Group interview and interviews	Multiple	Nurses (*n* = 7)	Supportive Care Needs Survey	Thus, we aimed to explore the feasibility and acceptability of the use of supportive care needs PROMs by colorectal cancer nurse specialists (CNS) in the delivery of supportive care to people with CRC receiving adjuvant chemotherapy.	Y	Y	Y	Y	Y	Y	Y	Y	Y	Y
Maguire et al. [44]	2008	UK	Sites (*n* = 6)	MM	Interviews and semi-structured questionnaires	Multiple	Nurses (questionnaire pre- *n* = 28, post *n* = 22, and interviews, *n* = 10)	Symptom questionnaire ASyMS	“This paper focuses on one of the secondary aims of the study, which was to explore the perceptions of nurses who participated in the study and who used ASyMS& to manage chemotherapy-related toxicity in clinical practice”. (p.382)	Y	Y	Y	Y	Y	N	Y	Y	Y	Y
Maguire et al. [45]	2015	UK	Clinical Centres (*n* = 5)	MM	Interviews and focus group	Lung	Health professionals (*n* = 13)	MSAS, Short Form and the Rotterdam Symptom Checklist Activity Subscale (ASyMS)	“Therefore, the primary aim of this study was to develop and explore the feasibility and acceptability of the ASyMS in patients with lung cancer receiving radiotherapy (ASyMS-R) and with clinicians involved in their care. A secondary aim was to explore changes in PROMs during the implementation of ASyMS-R, which could eventually inform the design and primary endpoints of future randomized controlled trials”. (E38)	Y	Y	Y	Y	Y	N	Y	Y	Y	Y
McCarthy et al. [46]	2016	Australia	Oncology Centres in hospitals (*n* = 3)	MM	Interviews and focus groups	Paediatrics	Health professionals (interviews *n* = 26 and focus groups *n* = 32)	Psychosocial Assessment Tool	“Study aims were to (1) investigate the feasibility (acceptability, brevity, simplicity) of administering the PAT2.0 psychosocial screener to parents following their child’s cancer diagnosis and to (2) examine oncology health-care professionals’ (HCPs) perspectives on the feasibility (acceptability availability, value, relevance) of the PAT2.0 screening tool in their clinical setting”. (p.364)	Y	Y	Y	Y	Y	N	Y	Y	Y	Y
Meldahl et al. [47]	2013	USA	Sample from various from practice settings	Qual	Focus groups	Multiple	Doctors (oncologists) (*n* = 20)	Various	“Therefore, our objectives were: (1) to explore oncologists’ understanding and attitudes toward PRO measures, and (2) to understand the impact of PRO data on clinical decision-making”. (p.725)	Y	Y	Y	Y	Y	N	U	Y	Y	Y
Noble-Jones et al. [48]	2019	UK	Clinic	Qual	Interviews	Genito-urinary	Health professionals (lymphoedema and urology) (*n* = 5)	Lymphoedema Genitourinary Cancer Questionnaire	“However, the LGUCQ had not been formally evaluated in an urooncology department to identify the benefits (or not) from the perspective of the patients and health professionals (urology and lymphoedema). Including acceptability” (p.5)	Y	Y	Y	Y	Y	Y	N	Y	Y	Y
Osborne et al. [49]	2014	UK	Organisations (*n* = 3) including, inpatient and outpatient and hospice settings	Qual	Interviews and focus group	Myeloma	Doctors, nurses and allied health (*n* = 6)	EORTC-QLQ-C30, MY24, POS, QOL q	“Aims of the present study are to (1) explore the issues important to QOL from the perspective of people with multiple myeloma, and (2) explore the views of patients and clinical staff on existing QOL questionnaires and their use in clinical practice”. (p.2)	Y	Y	Y	Y	Y	N	Y	Y	Y	Y
Semple et al. [50]	2018	Northern Ireland	Clinic	MM	Interviews	Oral/ Oro-phryngeal	Doctors (*n* = 2) and a nurse	UWQOLv4, Patient Concern Inventory	“Test the feasibility of using a disease- specific HRQOL tool and holistic item prompt list, for personalised identification and prioritisation of post- treatment concerns and issues, on a touchscreen computer, to promote patient empowerment and enablement, during routine surgical post- treatment follow- up clinic for oral and oropharyngeal cancer”. (p.3)	Y	Y	Y	U	Y	N	Y	U	Y	Y
Snyder et al. [51]	2013	USA	Cancer Centre in hospital	MM	Interviews	Multiple	Clinicians (*n* = 12) including medical oncologists and nurse practitioners	PatientViewpoint: Patient Reported Outcomes Measurement Information System + BR23 or the EPIC short-form.	“After developing a prototype website and conducting usability testing, [8] we sought to conduct the initial pilot-test of PatientViewpoint in practice to assess its feasibility and value. We examined the web tool’s use, usefulness, and acceptability”. (p.2)	Y	Y	Y	Y	Y	N	Y	U	Y	Y
Stover et al. [52]	2015	USA	Cancer Clinics (*n* = 3)	Qual	Questionnaires	Multiple	Doctors (*n* = 8) and nurse practitioners (*n* = 4)	Items from PRO-CTCAE and PROMIS and authors own	“Our primary objectives were the following: (1) solicit feedback from cancer clinicians and patients to develop a web-based screening system for securely administering and summarizing PRO measures for use during routine cancer care; and (2) pilot test the system during outpatient visits to evaluate cancer patients’ and clinicians’ perceptions of the acceptability and value of discussing PRO measures during clinical care”. (not provided)	Y	Y	Y	Y	Y	N	Y	U	U	Y
Sundberg et al. [53]	2015	Sweden	Hospitals (*n* = 2)	MM	Interviews	Multiple	Nurses (n = 8)	A standardized symptom and QoL questionnaire	“The aim of this study was to test the feasibility and acceptability of an interactive ICT-platform for smartphone use which collect and manage patient reported symptoms during radiotherapy for prostate cancer” (p.523)	Y	Y	Y	Y	Y	N	Y	Y	Y	Y
Taylor et al. [54]	2017	UK	Community-based palliative care	Qual	Interviews	Multiple	Doctor (palliative care) (*n* = 4), nurses (*n* = 7) and general practitioners (*n* = 4)	“PainCheck”: an electronic pain monitoring system	“To inform the development and implementation strategy of an electronic pain monitoring system, PainCheck, by understanding palliative care professionals’ needs when integrating PainCheck into routine clinical practice”. (p.661)	Y	Y	Y	Y	Y	U	Y	Y	Y	Y
Thayssen et al. [55]	2016	Denmark	Primary care	Qual	Interviews	Multiple	General practitioners (*n* = 11)	Patient questionnaire: ‘DT and ‘Impact Thermometer’ and problem list	“To examine how GPs experience to involve a short questionnaire, completed by patients’ prior to a consultation, when addressing the patients’ problems and needs. The aim is to contribute to the knowledge concerning the use of questionnaires as part of clinical cancer care in general practice”. (p.114)	Y	Y	Y	Y	Y	Y	Y	Y	Y	Y
Thewes et al. [56]	2016	Australia	Cancer Centres (*n* = 2) and Cancer Clinics (*n* = 2)	Qual	Interviews	Multiple	Clinicians (*n* = 10) including nurses, social workers, indigenous health workers and allied health	SCNAT-IP Supportive care needs assessment tool for Indigenous People	“This study describes patient and staff attitudes towards the acceptability and feasibility of the SCNAT-IP in routine care. Additionally, this study aimed to identify refinements needed to prepare the SCNAT-IP for use in clinical settings”. (p.2)	Y	Y	Y	Y	Y	Y	Y	Y	Y	Y
Velikova et al. [57]	2002	UK	Clinic in Hospital	MM	Interviews	Multiple	Doctors (oncologists) (*n* = 3)	EORTC QLQ-C30 + HADS	“This project was therefore undertaken to assess the feasibility of using computer-administered individual QL measurement in oncology clinics with immediate feedback of results to clinicians and to examine the impact of the QL information on the content of the medical consultations and on patient satisfaction with communication”. (p.52).	Y	Y	Y	Y	Y	N	Y	Y	U	Y
Velikova et al. [58]	2008	UK	Hospitals (2)	Qual	Focus groups	Multiple	Doctors (oncologists) (*n* = 16)	EORTC QLQ-C30	“The aim of this qualitative study was to explore what doctors and patients expect and want from routine measurement of QOL in clinical practice. The second aim was to generate ideas and produce recommendations of how to improve the questionnaires specifically for use in clinical practice”. (p.691)	Y	Y	Y	Y	Y	N	Y	Y	Y	Y

*Abbreviations*: *ASyMS* advanced symptom monitoring system, *BR23* Breast-cancer specific module from the European Organization for Research and Treatment of Cancer Quality-of-Life Questionnaire, *CR* clinician report, *DT* distress thermometer, *ESAS* Edmonton Symptom Assessment Scale, *EPIC-CP or – 26* expanded prostate cancer index composite for clinical practice, *EQ-5D* Euro-QoL EQ-5D health-related quality of life measure, *GP* general practitioner); HealthHUBTM)A Computerised assessment tool that includes the ESAS and the EQ-5D), *HRQOL* Health-related quality of life, *MM* Mixed-methods, *MSAS* memorial symptom assessment scale, *N* No, *PROMPT-Care* patient reported outcome measures for personalized treatment and care, *QOL or QL* quality of life, *Qual* Qualitative methods, *SAQ* symptom assessment questionnaire, *SCC* symptoms and concerns checklist, *SEIQoL-DW* schedule for the evaluation of the individual quality of life-direct weighting, *SFD* screening for distress, *U* unsure, *Y* Yes

The majority of studies (*n* = 24) focused on multiple cancer streams. Most study samples included a mixture of HPs, usually a combination of doctors and nurses, while others in addition included managers, administrators and allied health staff. Half of the studies (*n* = 17) used a mixed-methods design. Interviews were usually the sole qualitative method used (*n* = 22). Studies which used multiple qualitative methods paired interviews with focus groups (*n* = 3), with observations, (n = 1) or with a questionnaire (*n* = 1).

In some studies, the focus was on views about PROMs in practice, rather than a specific tool or measure (*n* = 6). The remainder of studies examined a range of PROMs; in which over half of patient reports were collected electronically (*n* = 18). The most common measures were: ESAS (*n* = 5); Problem Checklist (*n* = 4); Distress Thermometer (*n* = 3); and EORTC QLQ-C30 (n = 3) (see Additional file 2 for list of PROMs and abbreviations).

The included studies spanned PROMs development through to implementation; six studies reported on the development of the PROMs tool as well as evaluating it in practice. The majority of studies examined HPs’ views in relation to using PROMs in practice, either as part of a pilot or during roll-out (*n* = 28). While all of the studies focused upon HPs’ perceptions of PROMs, a number assessed acceptability as a key focus (*n* = 13).

### Quality of included studies

Studies were assessed as meeting between 6 and 10 of the quality criteria, with an average rating of 8.82. The majority of the studies reviewed (*n* = 30) were rated as satisfying at least 8 quality criteria. The item which received the fewest number of positive ratings was “Has the relationship between researcher and participants been adequately considered?” [18], with only 9 studies considered to have done so (see Table 1).

### Sentiment analysis

From the 34 primary texts, a total of 363 sentences relevant to HPs’ attitudes to PROMs were extracted. The mean sentiment score was found to be marginally positive at 0.08 (minimum = − 1.04, maximum = 1.43), with the highest density of sentences having a neutral sentiment. There was a greater positive density tail, demonstrating a higher number of positive comments. To gauge the meaning and context of the different sentiment scores here are some examples of sentences with a positive, negative and neutral sentiment score:

- “Well the training was excellent, the day that we had at the University was very informative, all the handouts were clear, there was plenty of time as well, and you could, there were loads of opportunities for questions, everything was absolutely splendid in terms of training.” This sentence had a positive sentiment score of 1.03.
- “I think of it more in terms of research of standardized questionnaires to evaluate the impact of interventions or peoples experience.” This sentence has a neutral sentiment score of 0.
- “So compared to the system that I’m used to, it seems cumbersome, it adds in too many other things to do to actually get to the people.” This sentence has a negative sentiment score of − 0.472

**Fig. 2:**
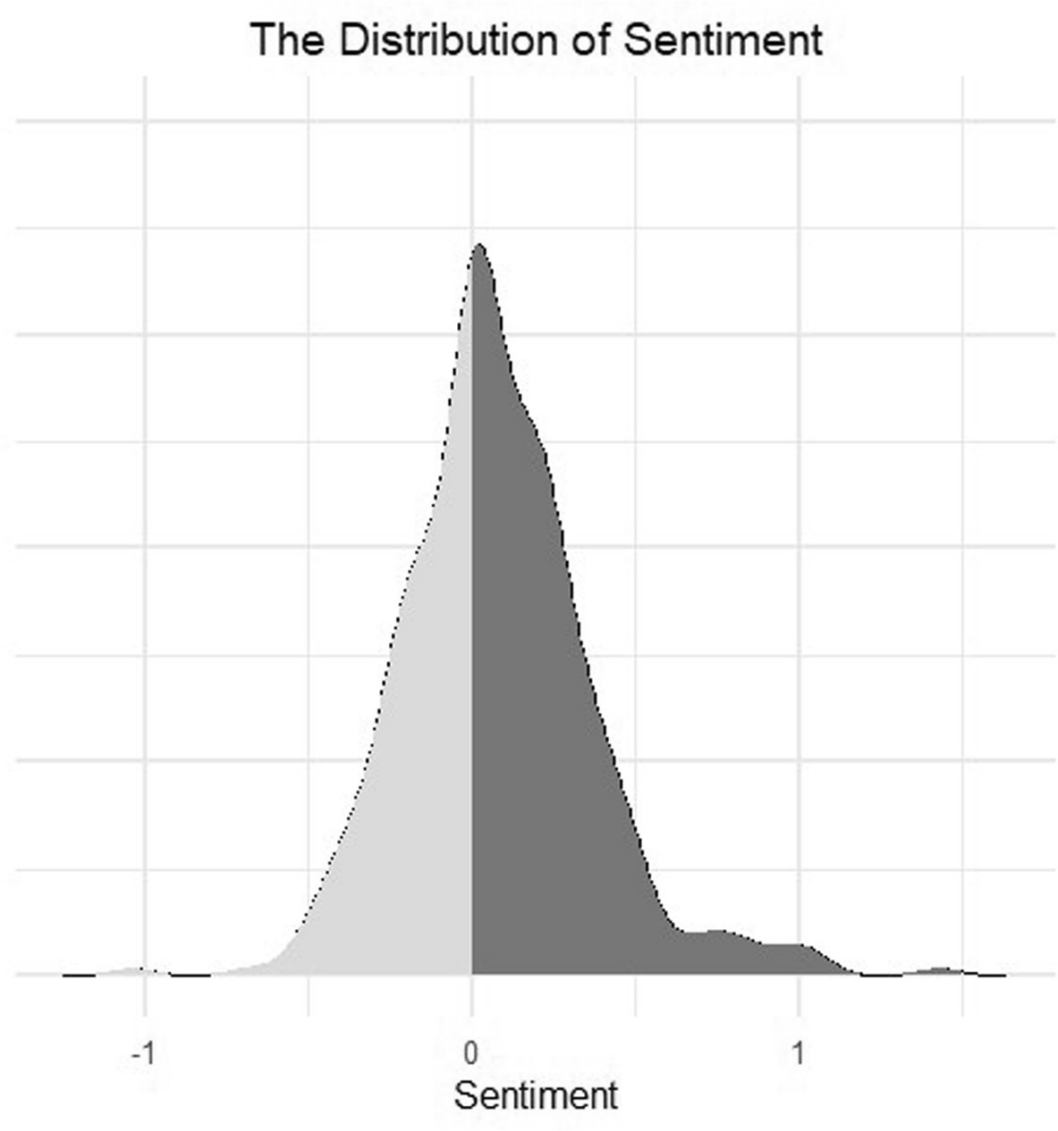
Sentiment scores for HPs’ attitudes towards PROMs

Figure 2 shows a density plot for the sentiment scores; there were a greater number of higher scoring positive sentiment sentences than negative ones. Positive comments often occurred when HPs were describing the usefulness of the benefits of the PROM, whereas negative comments were regarding feasibility of implementing PROMs, such as finding it to be time-consuming or cumbersome.

### Meta-synthesis

**Fig. 3:**
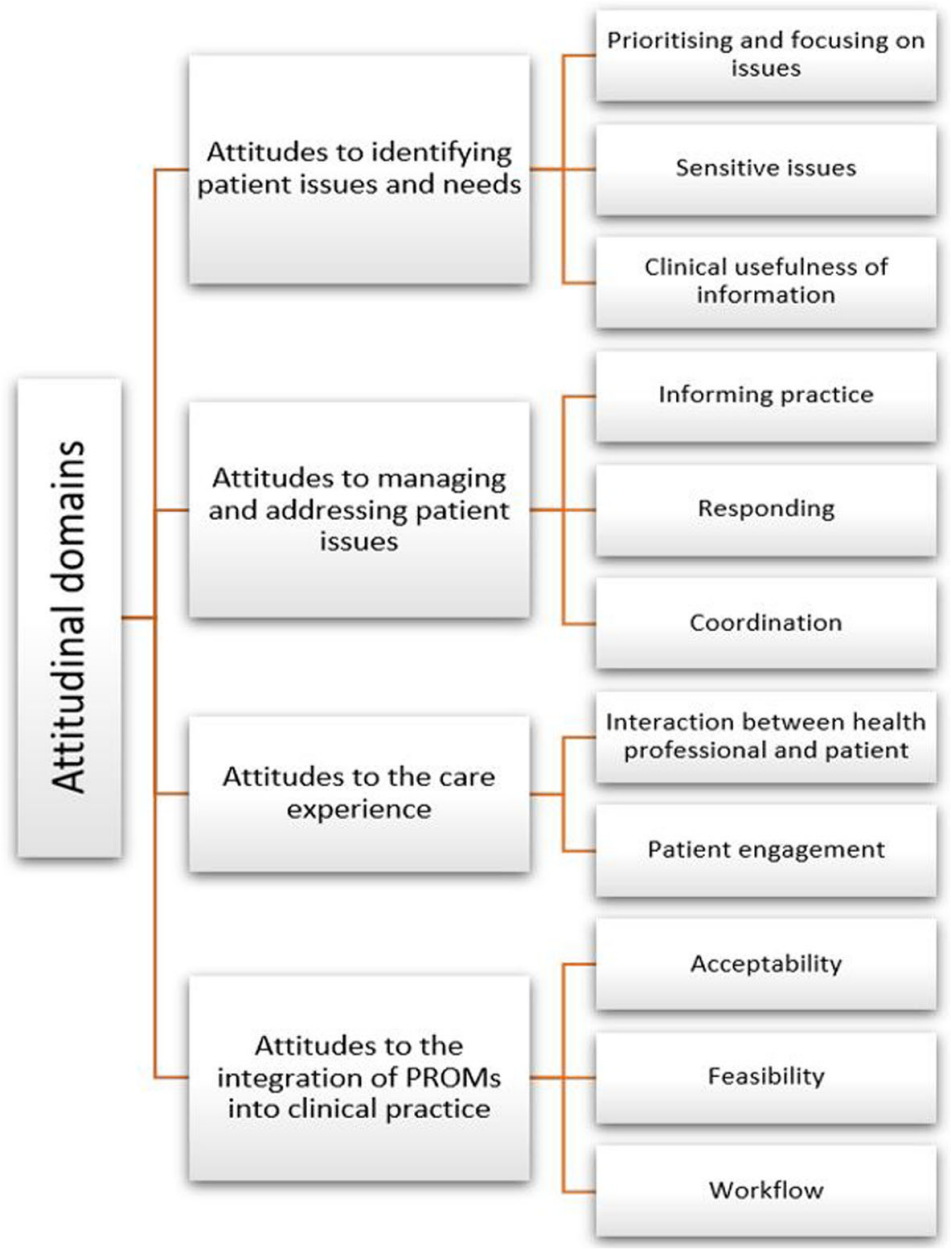
Themes identified regarding HPs’ attitudes towards PROMs

Four themes were revealed: HPs’ attitudes towards identifying patient issues and needs; managing and addressing patient issues; the care experience; and the integration of PROMs into clinical practice (see Fig. 3; Table 2).

**Table 2:** Themes identified in included studies

Studies	Attitudes to identifying patient issues and needs			Attitudes to managing and addressing patient issues			Attitudes to the care experience		Attitudes to the integration of PROMs into clinical practice			Barriers & Facilitators			
	Prioritising and focusing on issues	Sensitive topics	Clinical usefulness of information	Informing practice	Responding	Coordination	HP-Patient interaction	Patient Engagement	Acceptability	Feasibility	Workflow	Fit with practice	Value of PROMs	Capacity	Support
Absolom et al., 2011 [26]	✓		✓		✓	✓		✓	✓	✓	✓	✓			✓
Basch et al., 2005 [27]	✓		✓	✓	✓				✓						
Biddle et al., 2016 [28]				✓	✓	✓	✓	✓			✓	✓	✓	✓	✓
Brundage et al., 2019 [29]	✓	✓	✓	✓		✓	✓	✓			✓			✓	✓
Carolan and Campbell, 2016 [30]		✓	✓		✓	✓	✓		✓		✓		✓	✓	
Cox et al., 2011 [31]	✓		✓	✓			✓		✓			✓	✓		✓
DuBenske et al., 2008 [32]	✓		✓		✓				✓	✓		✓			
Gamlen and Arber, 2013 [33]	✓	✓	✓	✓			✓	✓	✓	✓		✓	✓		✓
Girgis et al., 2017 [10]	✓		✓		✓	✓	✓	✓	✓	✓	✓			✓	✓
Groff et al., 2018 [34]					✓	✓	✓	✓	✓	✓	✓	✓			✓
Handberg et al., 2018 [35]			✓	✓	✓		✓		✓		✓	✓			✓
Hubbard 2014 [36]	✓		✓	✓		✓			✓	✓	✓				
Jagsi et al., 2013 [37]	✓		✓		✓	✓	✓	✓	✓	✓	✓			✓	✓
Javid et al., 2017 [38]			✓						✓						
Kallen et al., 2012 [39]					✓				✓	✓	✓	✓	✓		✓
Kendall et al., 2013 [40]	✓	✓			✓				✓	✓		✓			
Kettis-Lindblad et al., 2007 [41]	✓		✓	✓					✓	✓	✓	✓	✓	✓	✓
Korzeniowski et al., 2016 [42]	✓		✓	✓	✓				✓		✓	✓	✓		
Kotronoulas et al., 2017 [43]	✓	✓	✓			✓			✓	✓	✓		✓	✓	✓
Maguire et al., 2008 [44]			✓		✓				✓	✓	✓		✓		✓
Maguire et al., 2015 [45]			✓	✓	✓			✓	✓	✓	✓	✓	✓		✓
McCarthy et al., 2016 [46]			✓	✓	✓	✓	✓	✓	✓	✓	✓		✓		
Meldahl et al., 2013 [47]			✓	✓	✓					✓		✓	✓		
Noble-Jones et al., 2019 [48]	✓	✓	✓	✓		✓		✓			✓				✓
Osborne et al., 2014 [49]	✓	✓	✓	✓		✓						✓	✓		
Semple et al., 2018 [50]	✓				✓	✓	✓	✓		✓	✓	✓			
Snyder et al., 2013 [51]	✓		✓	✓						✓			✓		
Stover et al., 2015 [52]	✓		✓	✓						✓	✓	✓	✓		✓
Sundberg et al., 2015 [53]				✓	✓			✓		✓	✓			✓	
Taylor et al., 2017 [54]				✓	✓	✓	✓	✓	✓	✓		✓	✓		
Thayssen et al., 2017 [55]	✓		✓				✓		✓				✓		
Thewes et al., 2016 [56]		✓	✓		✓	✓	✓	✓	✓			✓		✓	
Velikova et al., 2002 [57]	✓		✓	✓	✓	✓	✓			✓	✓	✓	✓	✓	
Velikova et al., 2008 [58]			✓	✓		✓	✓	✓		✓	✓	✓	✓		

#### Attitudes to identifying patient issues and needs

This theme captures HPs’ attitudes towards the identification of patient issues and needs using PROMs.

##### Prioritising and focusing on issues

PROMs were reported by HPs to be helpful for identifying a wide range of issues implicated in patient wellbeing. This included psychosocial [10, 26, 33, 51], and other quality of life issues which “often are overshadowed by attention to medical aspects of the disease and treatment” [41] (p284)

Where PROMs were completed prior to a consultation, this could aid HP preparation [27, 32, 33, 36] and promote careful reflection on the part of patients [52, 55]. PROMs were also a means to structure consultations and interactions, and aided to prioritise issues of importance [10, 29, 37, 40, 42, 43, 50, 57] (see also “Attitudes to the care experience”). Some studies [31, 42, 48, 49] noted that HPs also saw PROMs as prompting patients to identify and prioritise issues from their own perspectives: “Actually, it [using the questionnaire] meant that we talked about issues which we wouldn’t otherwise have touched upon because she hadn’t thought of it, and I usually don’t ask about it / … / it was actually quite important to her. It meant a lot to her” [55] (p117)

##### Sensitive issues

A few studies (*n* = 6) reported that PROMs could “facilitate open dialogue and discussion of sensitive topics” [29] (p776), related to issues that could be potentially upsetting or distressing [33, 40, 43, 48]. It was noted that this may vary by patient: “I do think it’s an important issue for patients, but obviously for some people it’s an embarrassing one to bring up … and it might be that you can’t solve the problem … but for some patients it’s a very simple ‘how many platelets do I need to have sex?” [49] (p11).

Distress or embarrassment may also be experienced by staff administering the PROMs [30, 33]. Only one study explored PROMs designed for Indigenous patients [56]; it highlighted the additional dimension of cultural sensitivity in the use of PROMs and professionals reported that the purpose-designed tool helped them to better connect with Indigenous patients [56].

##### Clinical usefulness of information

Studies revealed widely differing views as to what was gained from PROMs data. Some findings suggested that PROMs brought new information to the fore [33, 35, 41, 46, 48, 49, 55, 57], while others believed this information was already collected through other means [10, 26, 32, 42, 51]. The perceived meaningfulness of PROMs data was linked to the relevance of the items collected to the disease and the needs at different points over the patient’s journey [30, 37, 42–45, 52, 56, 57]; “patients’ problems vary during treatment and follow-up” [58] (p694). For some, the thresholds for alerts and detection of change over time were clinically useful in the identification of issues [31, 32, 36, 44, 58].

Some studies (*n* = 8) reported that HPs regarded PROMs data as clinically valuable and felt that the patient was the best judge of their symptoms, regarding their responses to be accurate and honest [29, 31, 42, 46–48, 55, 57]. As reported: “I trust you [the patient], you tell me when you’ve got a problem, you tell me how you’re getting on, here’s this piece of technology to enable you to do that” [31] (p678).

In contrast, Basch et al. [27] reported that clinicians thought that patients overstated their symptoms, or believed patients had difficulty distinguishing between the levels of severity [56]. On this theme, other studies reported that HPs wanted what they considered to be more objective, valid and reliable information about symptoms [32, 38].

#### Attitudes to managing and addressing patient issues

This theme addresses what happens with needs that are identified.

##### Informing practice

There were mixed views about whether PROMs would inform the practice of HPs. Some studies (*n* = 9) reported that PROMs helped HPs to ensure comprehensive coverage of issues and helped to refine the focus and streamline consultations [29, 41, 42, 48, 49, 51, 53, 54, 58]. HPs saw the potential for this to influence positively decision-making [27, 28, 31, 33, 42, 45, 49, 51, 52, 54]. In other cases, HPs felt PROMs information would have little impact on their practice or were uncertain about how much it influenced their patient management. Difficulties were also attributed to problems in interpretation of PROMs, to the timing of PROMs, the challenge of translating the data into the clinical domain and to a lack of guidance about how PROMs data might be integrated into decision-making [28, 35, 36, 47, 51, 57, 58].

##### Responding

PROMs were viewed as generating a range of responses in patient management. Response strategies included: intervention where issues were identified [26, 27, 32, 35, 46]; adoption of a more holistic management approach [40, 42, 45, 50]; modification of communication approaches [30, 32, 39, 47, 54, 56]; and the promotion of patient self-management [44, 53]. Study findings also raised the fear that PROMs could bring up issues for which no adequate response existed, particularly in relation to financial difficulties, psychological issues and fatigue [10, 28, 34, 37]:

I find it very hard to discuss finances with patients, especially when it comes to, “they say I'm not entitled to any benefits”, and I'm thinking, “well I can't do anything about that unfortunately”. I feel inadequate … because a patient could get a false sense from this thinking, “oh they can do something about it” … if it's on there [PROMs] … and you can't do anything about it [28] (p61).

##### Coordination

Coordination underpinned the management of PROMs. Prompting referral to specialists outside of the care team (e.g. allied health) was cited as a key benefit of using PROMs [29, 30, 37, 43, 46, 48–50, 57, 58]. Some studies highlighted the importance of clarifying roles and responsibilities in order to coordinate the management of care, especially in relation to psychological and emotional issues [30, 34, 36, 37, 54, 56]. For example, in a number of studies, oncologists and surgeons felt that nursing staff were better positioned to engage with psychosocial issues, and in some studies nursing staff agreed [10, 26, 28].

#### Attitudes to the care experience

Several studies reported on oncology professionals’ views of how PROMs may shape the experience of patient care.

##### Interaction between HP and patient

An often-cited benefit of PROMs was that it facilitated communication between the HP and the patient [29, 46, 50, 56, 57], allowing staff to get to know patients better [10, 34, 46, 50, 55]. For example:

the participants reported that SFD [PROM] helped them connect with patients by facilitating conversations and encouraging patients to communicate their concerns. This was seen through comments such as “I think it’s super helpful just to start conversations” and “It’s opened the conversation for other things that may have been going on in their lives that we weren’t seeing” [34] (p144).

In contrast, other studies raised the possibility that the use of PROMs might actually stifle rapport-building and diminish the “human touch” within the interaction [28, 30, 31, 33, 35, 37, 54, 58].

##### Patient engagement

PROMs are perceived as a means through which patients may more actively direct their own care [34, 37, 50]. HPs in some studies (*n* = 7) described the “empowering” facets of PROMs-supported engagement, noting it could promote the “voice” of patients in their care [29, 34, 37, 45, 48, 50, 56]. A few studies (*n* = 3) also noted the potential for PROMs to bring patients back into care when they had unresolved issues [10, 53, 54]. Some HPs expressed concerns that completing PROMs adds to the burden placed on patients, especially where they perceived patients to not be well enough [28, 46, 58]. Several studies (*n* = 5) cited practical and logistical barriers limiting the completion of PROMs by patients or their carers [28, 33, 37, 56, 58]. For example, HPs noted that there were a number of patient factors, such as language proficiency, literacy level, technological aptitude (where relevant) and residential location which may make it challenging for certain patients to participate in PROMS [26, 28, 37, 56].

#### Attitudes to the integration of PROMs into clinical practice

The attitudes clinicians hold towards the integration of PROMs into practice is explored in this final theme.

##### Acceptability

More than half of studies (*n* = 18) reported that HPs viewed PROMs to be acceptable, while noting that not all studies explicitly explored this. The acceptability was often linked with the perceived benefits described in the preceding themes, with an emphasis upon improving care and the experience for patients [10, 27, 31, 32, 34–37, 39–45, 55, 56]. Similarly, unacceptability derives from the challenges raised previously, such as, concerns about the relevance of a PROM, and issues relating to feasibility and the impact on workflow, as discussed below [26, 30, 33, 44, 46, 54].

##### Feasibility

Studies highlighted the importance of how PROMs were integrated with existing systems and practice. Where PROMs were electronic, integration within the existing management system was viewed to enhance feasibility [34, 36, 40, 46, 50–52], but it was important that the PROMs were easy to navigate and that professionals were equipped with the computer access, knowledge and skills they would need [10, 37, 39, 41, 45, 47, 54]. The introduction of PROMs also represented a shift in practice which needed to be carefully integrated in relation to the setting culture and as was noted as a key concern of staff, “alongside clinical priorities” [26, 33, 43, 44, 53, 57, 58]: “The participants indicated that there are ‘at least a hundred other priorities that compete with SFD [PROM] and these priorities have the potential to threaten the sustainability of the program’ and that SFD could fall off over time as other priorities emerge” [34] (p145).

##### Workflow

The implications of PROMs on workflow was widely explored in the studies reviewed. A frequently expressed concern was that the collection and management of PROMs could create additional demands on staff [ 26, 35, 42, 44–46, 57, 58]. This was particularly worrying for HPs who believed that this information to be already collected via other means.

While on the one hand fears were raised that PROMs would require an increased work and time commitment from professionals to address identified needs [10, 34, 35, 37, 41, 44, 50, 53], other HPs noted that PROMs presented opportunities for efficiency and time-svaing [29, 36, 39, 48, 50]. For example, where PROMs were regarded as focusing interactions (see “prioritising and focusing on issues”), this could enhance efficiency, as it would allocate attention to prioritised issues [29]. The introduction of PROMs into the existing flow of work was viewed as potentially challenging, but it was recognised that increasing familiarity would facilitate integration [43, 52].

Clarity concerning the roles and responsibilities of team members (e.g., who should manage psychosocial issues) [28], emerged as an important issue for managing workflow [28, 30]. There is also a recognition that certain key personnel may be over-extended as a result of PROMs: “Oncologists perceived the CNS [Clinical Nurse Specialist] as a ‘crutch’ for patients but also appreciate they are a stretched resource” [26] (p604).

### Barriers and facilitators to the uptake of PROMS

A number of factors were found to impede or promote the adoption and use of PROMs. These centred around: their fit with existing practice; how PROMs were valued; the capacity of professionals to respond to PROMs; and the support in place.

#### Fit with practice

Perceptions that PROMs would not align with or would disrupt existing practice were identified as a barrier to uptake, especially where this was viewed as an additional task on top of other competing demands [26, 32, 34, 35, 40, 41, 56, 57]. Embedding PROMs within the existing systems, frameworks and practice could act as a facilitator: “For the future, the introduction and implementation of an assessment tool should be embedded within the total assessment process” [33] (p801).

Guidelines for the use of PROMs in practice could facilitate integration [28, 52, 57]. For electronic PROMs, a lack of fit within the existing patient management system formed a barrier (e.g. if it operated separately to this) [31, 32, 34, 40, 45, 52], while full embedding within the patient management system and ease of use were enabling factors [32, 34, 39, 40, 42, 47–50, 58].

#### Value of PROMs

HPs’ perceptions about the value of PROMs may shape their uptake and use. A negative perception of the clinical usefulness of PROMs may be a barrier, while positive perceptions may promote use. The perceived relevance, specificity and evidence for the PROMs introduced is therefore key [28, 31, 33, 41–47, 49, 51, 52, 57, 58]. Another dimension of value related to whether HPs viewed PROMs to positively impact patients, with a lack of perceived benefit forming a barrier [30, 39, 54, 55, 58].

#### Capacity

Barriers emerge where professionals did not feel there was capacity to respond to PROMs for a range of reasons including self-efficacy, access to specialised knowledge, management arrangements within the team or the nature of the issues raised [10, 28–30, 37, 41, 43, 53, 56, 57]: Once you’ve had the interview and you say, “right, okay, I’ll go and make these phone calls”, … “right, ok, I’ve got to do this, I’ve got to do that”, and no added time was given. [It’s] the aftermath as well … if they’d allowed us more time, I think it could have been more effective [28] (p63).

#### Support

Training, practice opportunities and ongoing support would help to facilitate the adoption of PROMs [10, 26, 28, 29, 31, 33, 34, 39, 41, 43–45, 48, 52]. Additionally, appreciation on the part of managers about the impacts of PROMs and the involvement and “buy in” from front-line staff could support implementation [34, 35, 37].

## Discussion

We systematically reviewed qualitative evidence to enhance understanding of HPs’ attitudes towards PROMs in oncology services, and to gain insight into the barriers and facilitators for their adoption and use. Our meta-synthesis identified four key attitudinal domains: identifying patient issues and needs using PROMs; managing and addressing patient issues; the care experience; and the integration of PROMs into clinical practice. Key considerations for implementation of PROMS include: the fit with existing practice; how PROMs are valued; the capacity of professionals to respond to PROMs; and the supports in place.

PROMs have received increased attention from oncology researchers [4, 5]. Overall, the qualitative evidence is of good quality and captured recent findings, with half of the studies having been published from 2015 onwards. The inclusion of qualitative content from mixed-method research, where appropriate, facilitated examination of findings from study designs that appear to be often employed in research on the development and piloting of PROMs. The majority of studies are based in North American and European contexts, and encompass a range of service settings where PROMs may be applied, as well as presenting the views of various professional groups involved in their use. In many cases, studies included well-established patient measures, but also included views about PROMs use as a general concept.

The overall sentiments from the opinions of HPs towards PROMs was mostly neutral, with a more positive tail compared to negative densities. Consistent with the literature, many HPs believe PROMs support communication with patients; enabling them to know their patients better [4, 5, 43] but there are concerns that PROMs could negatively impact these relationships by diminishing the “human touch” in the care process [15]. In relation to sensitive or embarrassing issues for patients, PROMs were viewed as both potentially facilitative and potentially upsetting; this reflects previous findings [3, 8]. Interestingly, a few studies reported that embarrassment or distress may also be experienced by HPs using PROMs [30, 33]. One study included in-depth considerations of the cultural dimensions of PROMs, in which largely favourable views were expressed by HPs about the use of culturally appropriate PROMs designed for care of patients from Australian Indigenous communities [56]. We would advocate for further exploration of PROMs development and use in the care of patients from culturally and linguistically diverse communities, especially as our analysis noted that HPs viewed patients’ language proficiency as a potential impediment to meaningful participation.

Many HPs perceived PROMs as facilitating the identification of patient issues, including non-medical concerns such as psychosocial and quality of life issues [3, 4, 12, 15]. Some HPs see PROMs as empowering patients by encouraging them to give voice to issues they experience as significant and facilitating their capacity to play a more active role in their care, including through self-management (c.f. Howell et al. [3]). However, staff also gave careful thought to logistical and practical barriers that patients may face in reporting PROMs, and to the burden this could place on patients. The capacity for these measures to positively affect patients’ care was a key element of HPs’ appraisal of the value of PROMs; it is a likely a critical determinant of the active uptake of PROMs in practice.

The nature and timing of the PROMs data to be collected are important. In alignment with previous reports, HPs placed value on the collection of data which was: novel (i.e. avoiding duplication); relevant, containing clear indicators of clinical significance; appropriate for the specific cancer; and collected at significant points in the patient journey. These valuations were interwoven with HPs’ views about how these PROMs data would be subsequently used in practice. Perceptions of poor clinical utility could form an implementation barrier. HPs recognised the potential to promote earlier intervention and more holistic approaches to care but were concerned where they felt unable to adequately respond to the issues identified (e.g., issues for which feasible solutions were unavailable).

Some studies reported that HPs were concerned that some patients may have difficulty distinguishing levels of severity and therefore they desired more objective measures [27, 32, 38, 56]. A clinician with these concerns, overall or for a specific patient, may find it challenging to know how to best proceed. This mix of positive views and negative fears is perhaps not surprising, as previous reviews have not been able to establish conclusively the impact of PROMs on corresponding clinical actions, such as an increase in appropriate referrals or changes to patient management plans [5, 8, 13].

Howell et al. [3] observed that the relationship between the clinical communication of PROMs data and health outcomes is complex. This meta-synthesis of professional end-users’ accounts can contribute to deepening understanding surrounding the potential for disjointedness. Staff members’ views about their capacity to respond to PROMs data are crucial: they need the skills to engage with patients about needs identified by PROMs, including the skills and confidence to discuss sensitive subjects; they need to be comfortable that they have sufficient time to respond, given competing priorities in their workload; and they need to have referral pathways for patients whose needs require additional time or expertise. As coordination may bring challenges, the clear delineation of roles and responsibilities associated with responding to PROMs data within care teams may help to optimise a response. Further, recognition of any additional time that may be needed for the management of PROMs data, including accounting for additional time during the initial learning curve could facilitate active uptake. This may be especially important where HPs need to develop additional technological skills.

Findings from Chen et al.’s [5] systematic review suggest that the reporting of data alone may not be sufficient, but that positive changes in patient management could be more probable where PROMs collection is integrated into care planning. Our findings echo this principle and point to several priority areas for integration. From the accounts of HPs, PROMs use could be facilitated where it is embedded within the existing patient management system, and where staff are able to navigate this with ease. In this regard, the electronic collection of PROMs may have a distinct advantage. It was also deemed important that PROMs be incorporated within existing practices (e.g. consultations or assessment practices) and this could optimise efficiency. Providing staff members with guidelines for the use of PROMs in clinical practice may also be beneficial. Consistent with the literature [4, 5, 13, 15], our analysis would suggest that implementation efforts could be enhanced by the provision of staff training and ongoing support, establishing “buy in” from front line staff and appreciation on the part of managers about how PROMs may impact clinical practice. However, many of the issues raised such as coordination, workflow and clinical utility, which form negative HP attitudes are vital to tackle alongside these recommendations to improve implementation prospects.

### Limitations

The search was restricted to four databases, included only English language records and excluded grey literature. It is therefore possible that relevant studies may have been overlooked. We deemed it informative to include qualitative findings from mixed-method designs as well as qualitative studies. This resulted in the inclusion of studies with a range of methods and varying proportions of qualitative research (e.g. the qualitative content might have only represented a minority component of a mixed-method study). We sought to manage this through applying a criterion for studies to have analysed and reported on qualitative content separately, in addition to employing a uniform quality assessment procedure. The qualitative evidence comes from studies conducted in diverse health service contexts, including multi-centre studies. As such, the general concerns about de-contextualisation and over-extrapolation in qualitative meta-synthesis required careful consideration in the process of synthesis and analysis [59, 60]. We purposefully selected a strategy that involved an iterative cycle of engagement with the extracted data as part of the analysis process and was grounded in an approach successfully used with a large data set of qualitative studies [24].

Our study excludes the attitudes of patients and thus may miss some important PROMs implementation information. However, following initial scoping of the relevant literature, we deemed it necessary to narrow the scope so to ensure sufficiently comprehensive engagement with the substantial and diverse HP evidence base. Similarly, we would consider patient attitudes towards PROMs in oncology to warrant dedicated and in-depth study. Further, it is a limitation for the sentiment analysis that results could only be obtained from primary text available in the manuscripts that were synthesised and not all the qualitative data that were collected in these studies. Most qualitative manuscripts present only representative quotes, and often these consist of positive and negative representatives. Our neutral finding in the sentiment analysis may be due to this limitation, where a balanced representation was provided from the papers.

## Conclusion

The use of PROMs in oncology services may offer a valuable tool to help identify and manage patients’ needs over the course of their cancer journey. This paper advances existing understanding by reporting on a body of evidence which captures the sentiments and perspectives of those end-users critical to the adoption of PROMs in oncology practice. The findings articulate important pre-conditions, from the perspectives of HPs, for the successful implementation of PROMs; providing insights for policy-makers and services involved in rolling out these initiatives. Examination of the evidence points to several future research directions. Further exploration of the potentially empowering dimension of PROMs use could begin with a meta-synthesis of the qualitative evidence concerning oncology patients’ experiences with PROMs. The accessibility of PROMs for patients, including those from culturally and linguistically diverse communities would be valuable to synthesise. Implementation efforts could be enhanced through greater knowledge of the resources and care processes needed to support the translation of collected PROMs data into corresponding actions, to the benefit of patients.

## Supplementary information

**Additional file 1.** PRISMA Checklist.

**Additional file 2.** List of PROMs abbreviations.

## Data Availability

Articles included in the analysis are cited in the reference list.
