# Supplementary material for: What are the attitudes of health professionals regarding patient reported outcome measures (PROMs) in oncology practice? A mixed-method synthesis of the qualitative evidence
Source: BMC Health Serv Res. 2020 Feb 10;20:102. doi: 10.1186/s12913-020-4939-7 (PMC7011235; doi:10.1186/s12913-020-4939-7)
Supplement: Supplementary file 2 — Additional file 2. List of PROMs abbreviations. [file 12913_2020_4939_MOESM2_ESM.docx]

**Additional File 2: List of PROMs abbreviations**

| ASyMS | Advanced Symptom Monitoring System |
| --- | --- |
| BR23 | Breast-cancer specific module from the EORTC QLQ-C30 |
| CR | Clinician Report |
| DT | Distress Thermometer |
| EORTC QLQ-C30 | European Organization for the Research and Treatment of Cancer Quality of Life Questionnaire |
| ESAS | Edmonton Symptom Assessment Scale |
| EPIC-CP or -26 | Expanded Prostate Cancer Index Composite for Clinical Practice |
| EQ-5D | Euro-QoL EQ-5D health-related quality of life measure |
| GP | General Practitioner |
| HealthHUBTM | A Computerised assessment tool that includes the ESAS and the EQ-5D |
| HRQOL | Health-related quality of life |
| MM | Mixed-methods |
| MSAS | Memorial Symptom Assessment Scale |
| PROMPT-Care | Patient Reported Outcome Measures for Personalized Treatment and Care |
| QOL or QL | Quality of life |
| Qual | Qualitative methods |
| SAQ | Symptom Assessment Questionnaire |
| SCC | Symptoms and Concerns Checklist |
| SEIQoL-DW | Schedule for the Evaluation of the Individual Quality of Life-Direct Weighting |
| SFD | Screening for Distress |
